# What do young Australian women want (when talking to doctors about contraception)?

**DOI:** 10.1186/s12875-017-0616-2

**Published:** 2017-03-15

**Authors:** Denisa L. Goldhammer, Catriona Fraser, Britta Wigginton, Melissa L. Harris, Deborah Bateson, Deborah Loxton, Mary Stewart, Jacqueline Coombe, Jayne C. Lucke

**Affiliations:** 1 Australian Research Centre in Sex, Health and Society, La Trobe University, Melbourne, Victoria Australia; 2 Social Research Unit, WorkSafe Victoria, Melbourne, Victoria Australia; 3 School of Public Health, University of Queensland, Herston, QLD Australia; 4 Research Centre for Generational Health and Ageing, University of Newcastle, Newcastle, NSW Australia; 5 Family Planning NSW, Sydney, NSW Australia; 6 The University of Sydney Discipline of Obstetrics, Gynaecology and Neonatology, Sydney, NSW Australia

**Keywords:** Contraception, Communication, Patient-provider Interaction, Young Women, Reproductive Health, Qualitative Research

## Abstract

**Background:**

Access to most contraceptives in Australia requires a prescription from a doctor, and it has been shown that doctors can influence women’s decision-making with respect to contraception. However, little research has documented how women experience their interactions with doctors within the context of a contraceptive consultation. Understanding such experiences may contribute to our knowledge of factors that may influence women’s contraceptive decisions more broadly.

**Methods:**

We report on findings from the Contraceptive Use, Pregnancy Intentions and Decisions (CUPID) survey of young Australian women, a large-scale longitudinal study of 3,795 women aged 18–23 years. We performed a computer-assisted search for occurrences of words that indicated an interaction within the 1,038 responses to an open-ended question about contraception and pregnancy. We then applied a combination of conventional and summative content analysis techniques to the 158 comments where women mentioned an interaction about contraception with a doctor.

**Results:**

Our analysis showed that women desire consistent and accurate contraception information from doctors, in addition to information about options other than the oral contraceptive pill. Some young women reported frustrations about the choice limitations imposed by doctors, perceived by these women to be due to their young age. Several women expressed disappointment that their doctor did not fully discuss the potential side-effects of contraceptives with them, and that doctors made assumptions about the woman’s reasons for seeking contraception. Some women described discomfort in having contraception-related discussions, and some perceived their doctor to be unsupportive or judgmental.

**Conclusions:**

Both the content and the process of a contraceptive consultation are important to young Australian women, and may be relevant contributors to their choice and ongoing use of a contraceptive method. These findings provide useful insights into aspects of the patient-provider interaction that will enhance the efficacy of the contraceptive consultation. It is recommended that doctors adopt patient-centred, shared decision-making strategies to support women in making choices about contraception that suit their individual circumstances. We also acknowledge the need to involve other health care providers, other than doctors, in educating, informing, and assisting women to make the best contraceptive choice for themselves.

## Background

Most sexually active Australian women of reproductive age who want to avoid pregnancy use contraception [1]. Several forms of hormonal and non-hormonal contraception are available: condoms, the diaphragm, the oral contraceptive pill (OCP), the vaginal ring, long-acting reversible contraception (LARC) options including implant, injection, and intrauterine methods, as well as the over-the-counter levonorgestrel emergency contraceptive (LNG-EC) pill [2]. Short-acting methods such as the OCP have lower effectiveness than LARC [3, 4]; yet despite the availability of numerous, highly effective LARC methods and recent clinical recommendations encouraging the uptake of such options [5, 6], the most common contraceptive method used by young Australian women remains the OCP [7–9]. While the OCP is effective, many women find it difficult to use consistently, which leads to high discontinuation rates [10]. In addition, there is a high rate of induced abortion in Australia [11], suggesting that contraception non-use, ineffective use, and failure remains a significant problem. Previous research has highlighted that although both women and men acknowledge the need for contraception to prevent unintended pregnancy, they generally lack knowledge about the range of methods available to them and how to use their preferred method correctly [12].

Aside from the condom, LNG-EC pill, and fertility awareness-based methods (i.e. diaphragms, withdrawal, and natural family planning), access to other contraception methods in Australia necessitates a doctor’s prescription, obtained most commonly through consulting a general practitioner or family planning clinic. Doctors working in such settings therefore typically represent the first point-of-contact for young people seeking sexual health and contraceptive advice and may have a substantial influence on young women’s decision-making with respect to contraception. Indeed, a US cohort study of 1,387 women aged 15–24 years who were starting a hormonal contraceptive found that despite the majority of participants reporting that they had selected their chosen method for themselves, over half also reported that they selected the method because of what their healthcare provider had told (or had not told) them [13]. Comparably, a qualitative investigation of young women’s contraceptive choices before and after seeing a provider at a family planning clinic found that just under a quarter of participants who reported not desiring a hormonal or LARC method prior to the interaction ultimately selected such a method after discussing LARC methods with the provider [14].

Best practice in contraceptive care across several countries including Australia, the UK, and the US has moved away from a doctor-centred model of care toward a patient-centred care model, which conceptualises patients as consumers who are informed and engaged when making decisions about their own health [15]. To make good decisions about their own health however, patients need to have access to reliable, comprehensive information [16]. To make good decisions about contraception, women must be aware of the available options and be able to weigh up the benefits and disadvantages of various methods for their own particular situation. Shared decision-making, a core component of the patient-centred model of care, requires the woman and doctor to reach a shared understanding of the woman’s situation and preferences [17]. In the process of shared decision-making, the clinician’s role is “to help patients become well-informed, help them develop their personal preferences for available options, and provide professional guidance where appropriate” ([18], p. 271). As this ‘position description’ suggests, the issue is not as simple as providing accurate information in a unidirectional manner; the clinician also has to interpret information that the patient provides and deliver advice within a bidirectional interactional system. Recent research suggests that women are more likely to contribute to decisions about contraception than they are about their general healthcare [19], with contraceptive consultations found to be most effective when the woman’s concerns are correctly understood and addressed by the clinician [20].

Through documenting the decisions that women make following a contraceptive consultation, the literature reviewed above evidences that healthcare providers- particularly doctors- can influence a woman’s contraceptive choices. In order to understand the factors that may influence these choices more broadly, it is also important to appreciate women’s interactional experiences with a doctor in the context of a contraceptive consultation. For example, an interview-based qualitative study of women’s perceptions of the contraception consultation in the UK found that women felt judged on social as opposed to medical grounds when consulting doctors about contraception, with doctors routinely dismissing or disregarding women’s reports of adverse side-effects [21]. Women’s subsequent contraceptive choices were evidently influenced by these interactions, as well as their trust in the healthcare provider. Furthermore, a recent Australian study exploring how women obtain and use contraception (and the barriers to doing so) found that young women reported dissatisfaction with the amount of information doctors provide about contraception in addition to negative interactions with healthcare providers when accessing contraception [22].

In order to understand what young (Australian) women want- and do not want- when interacting with a doctor in the context of a contraceptive consultation, we specifically aim here to qualitatively document how women perceive and report on their interactions with a doctor about contraception. Such findings contribute to the broader question of what factors may influence the decisions women make about contraception use and method choice. Throughout this paper, the term ‘doctor’ is used to refer to a doctor of any speciality who provides contraceptive care to women, and is not limited to describing general practitioners.

## Methods

### Study design and participants

The data we report on here were drawn from the 3,795 women who responded to the Contraceptive Use, Pregnancy Intentions and Decisions (CUPID) study; a longitudinal, population-based cohort study of young Australian women aged 18–23 years. The overall aim of this study was to examine factors influencing contraceptive use and unintended pregnancy among this cohort using three waves of online self-report surveys conducted at six-monthly intervals. Aspects of establishing the study and recruitment for the online survey are outlined in detail elsewhere [23]. In brief, women were recruited through media coverage, attendance by the researcher team at face-to-face events, social media (including paid Facebook advertising), Family Planning clinics, and word of mouth. All women aged between 18–23 years who were living in Australia at the time of the survey were eligible to participate. The cohort recruited was demographically representative of young Australian women, with the exception of an over-representation of high school completers [23]. The data reported here are from the baseline survey.

Several open-ended, free-text questions were included as part of the CUPID survey to allow for an expanded understanding of women’s experiences. Analysis of free-text comments from surveys can provide a deeper understanding of survey topics, raise issues and reveal conceptual relationships not previously considered important by researchers [24, 25]. In this analysis we examined women’s reports of the interactions they had with a doctor about contraception from open-text responses to one general question about contraception and pregnancy, located at the end of the survey: *Is there anything else you would like to tell us about contraception (protection), your plans for pregnancy, or your experience of pregnancy?*

### Data collection and analysis

Of the 3,795 participants in the survey, 1,038 (27.4%) answered the open-ended question of interest. As the focus of this article was to examine women’s reports of interactions about contraception, data analysis began with computer-assisted searches for occurrences of words that indicated an interaction. That is, we extracted free-text comments that contained one of the following words and their derivatives: *talk*; *told*; *tell*(*s*) (*ing*); *speak*(*s*) (*ing*); *discuss*(*es*) (*ing*) (*ed*); *state*(*s*) (*d*); *mention*(*s*) (*ing*) (*ed*); *ask*(*s*) (*ed*) (*ing*); *hear*(*d*); *suggest*(*s*) (*ing*) (*ed*), and so forth. We then examined the extracted comments in order to seek understanding of the context within which these terms were used, applying a combination of conventional and summative content analysis techniques to comments where women mentioned an interaction about contraception with a doctor (the most commonly identified group). The extracted comments ranged in length from a few words to multiple sentences.

Using qualitative content analysis methods allows researchers to interpret the content of text data through the systematic process of coding and identifying themes [26]. Summative content analysis involves the identification of key content followed by interpretation of the underlying context, while conventional content analysis involves the creation of an efficient number of coding categories that represent similar meanings and are derived directly from the text data [26]. We have integrated these two approaches to content analysis because they align with our research aim of capturing the participants’ unique perspectives without the imposition of preconceived categories or theoretical perspectives.

Three of the authors (DG, CF, and JL) immersed themselves with the data through reading the comments extracted in the initial phase of analysis closely and repeatedly. They then highlighted words or sections of the text that appeared to capture women’s meanings within the context of talking with a doctor about contraception, inductively generating coding categories. The unit of analysis was the portion of text in which the woman described the interaction; typically this was a brief portion of text, at most a sentence or two. As this process continued, coding categories that represented similar concepts were organised into meaningful clusters, and labels that captured the key theme/s of each cluster were developed by the first author. To determine trustworthiness of the themes, a process of reiterative feedback and discussion among all authors was undertaken until consensus was reached.

## Results

Of the 1,038 comments gathered by the final question in the survey, 20.4% (*n =* 212) contained a term that flagged a reported interaction. In comparison with the whole cohort, a larger percentage of the women who answered the final free-text question were engaged in full-time study and living in urban areas. They were also more likely to have finished secondary school, although not more likely to have completed tertiary education. Table 1 compares the demographic information for all participants, those who answered the final question, and those who reported on an interaction in their final answer.

**Table 1:** Demographic characteristics of all CUPID participants and study data subsets

	All CUPID participants N (%)	Answered Final Question N (%)	Reported on Talk N (%)
Total number	3795 (100%)	1038 (27.4%)	212 (20.4%)
Urban postcode	2415 (63.6%)	694 (66.9%)	158 (74.5%)
Median age (years)	20.7	20.8	20.9
Education			
• Did not finish secondary school	345 (9.1%)	88 (8.5%)	14 (6.6%)
• Graduated secondary school	1673 (44.1%)	450 (43.4%)	98 (46.2%)
• Graduated tertiary education	1692 (44.6%)	495 (47.7%)	95 (44.8%)
Work status			
• Full time study	1831 (48.2%)	509 (49.0%)	122 (57.5%)
• Part time work	1734 (45.7%)	494 (47.6%)	98 (46.2%)
• Full time work	910 (24.0%)	237 (22.8%)	51 (24.1%)

Of the 212 women who reported an interaction about contraception in their comments most (75%, *n =* 158) mentioned talking to a doctor, 17% (*n =* 37) reported talking to their sexual partners, 9% (*n =* 20) to family members, primarily mothers, 8% (*n =* 17) reported interactions with friends, and 14% (*n =* 29) talked to someone else, for example a pharmacist or teacher. Some women (*n* = 49) reported speaking to more than one of these people.

The data presented here represent interactions that women specifically had with doctors during consultations about contraception, capturing overall themes related to both the content and the process of such interactions. These emergent themes and respective subthemes extracted from the data are presented in Table 2, and are described in detail below alongside illustrative quotes from participants. In presenting response extracts, we have indicated the participant’s age and geographical location (urban, regional, or remote^1^). Words that have been omitted or inserted for contextual clarification are indicated by an ellipsis or in roman script encircled by square brackets, respectively.

**Table 2:** Overall themes and key findings extracted from the data, *N =* 212

Theme	Key findings
Women’s perspectives on the content of contraceptive discussions with doctors: what women want	• Consistent and accurate advice about contraception • More information about contraception options • No discrimination based on young age of the woman • Comprehensive information regarding the potential side-effects of the chosen method • Specific consideration of contraception options and related issues for women with complex medical profiles (e.g. women with PCOS or endometriosis)
Women’s perspectives on the process of contraceptive discussions with doctors: what women experience	• Difficulties speaking to their doctor about contraception, due to e.g. their young age and/or personal discomfort • Perceptions of their doctor as unsupportive or even judgmental

### Women’s perspectives on the content of contraceptive discussions with doctors: what women want

When reporting on the content of patient-provider interactions, young women overwhelmingly described a desire for their doctor to present consistent and accurate advice related to contraception, and highlighted their difficulties in accessing such information at times. In some cases women reported that they had received contradictory information from different doctors, leading to frustration and uncertainty about their contraception options:

*I have had extreme difficulty finding a form of contraception that works for me and am often told conflicting things by different doctors.* (21 year old urban resident)

*I usually see two different doctors to gain a second opinion. It is unhelpful when they say several different things and* [are] *essentially contradicting each other.* (22 year old urban resident)

Women similarly reported dissatisfaction around the often limited contraception options that their doctor had discussed with them, indicating that they would like to have received more information about other eligible options. In particular, our analysis suggested that- in some cases- information regarding options other than the contraceptive pill (‘the Pill’) was not provided by doctors:

*Often the Pill has been presented as the only option/solution by doctors. I have done my own Internet research about different types of contraceptives like the arm implant* [contraceptive implant^2^]*. I wish I had been given more information about this and other options by a medical practitioner.* (23 year old regional resident)

*I want the best for my body and don’t want to disrupt it too much, and I have suggested* [to doctors] *in the past that I want to use an alternative to the Pill (not condoms either) and the doctors have not given me options.* (23 year old urban resident)

Furthermore, some women reported being exasperated due to a perception that their doctor was limiting their contraceptive choices as an apparent reaction to their (young) age. Some women thought that doctors perceived them to be ineligible for certain contraception methods owing to their age:

*I feel that some doctors are biased* [against] *giving young women longer-term contraceptive methods (Mirena IUS* 3 *) due to their age and the idea that they may want children soon…the Mirena IUS sounded like a good option for me, but I had to try multiple type of contraceptive pills before my doctor would agree to insert it because it was considered a “last resort” for women my age.* (22 year old regional resident)

Women reported on the difficulties of obtaining comprehensive knowledge with respect to their chosen contraception method, and in particular around gaining an accurate understanding of the potential side-effects of the method:

*I was using Implanon* ^2^ *and after two successful years I began bleeding daily for about three months. I didn’t know what was happening and my doctor couldn’t tell me and just advised me to get a new one. Another doctor told me later that it can 'wear off'. If this is the case, people should be notified- most importantly doctors, so they can advise correctly. Public knowledge can only go so far; professionals need to keep up to date.* (22 year old urban resident)

*I changed doctors (several years ago). My new doctor informed me of how easily available the NuvaRing* [vaginal ring^4^] *was - my last doctor never mentioned it as an option and I was under the impression that it was difficult to access in Australia at the time. Changing doctors really changed the quality of my health regarding contraception.* (23 year old urban resident)

Specifically, the potential side-effects of a chosen contraception method were highlighted by women as a priority discussion point with their doctor, which reportedly did not always occur- or else was not perceived as satisfactorily thorough:

*When I asked a doctor about the Implanon rod* ^2^, *I wasn't really given information. She just asked if I was sure and gave me a script. I've had the rod for a few months now, and whilst the convenience of not having to worry about taking a pill every day is good, I wish I had been told about the side-effects. I've had weight gain, acne, mood swings and most annoyingly, month-long periods. If I had been given this information, I would have just stayed on the Pill or looked into another method.* (20 year old urban resident)

*I wish there was more information on the adverse reaction you can have with the Pill or hormonal contraceptive and what other options I can take…any information I can get on this subject would be more than what I am currently receiving.* (22 year old urban resident)

Women with more complex medical profiles- such as those experiencing polycystic ovarian syndrome (PCOS), endometriosis, or a high body mass index (BMI)- similarly highlighted the importance of discussing their contraceptive options with their doctor, emphasising the need to talk about possible side-effects. Some also indicated that their choice to use a particular contraceptive method was not necessarily influenced by its reproductive action, but rather by non-contraceptive benefits:

*After having endometriosis and PCOS for years I think doctors and nurses need to be more educated in what contraceptive works for these issues. I've been prescribed methods of contraceptive in the past that have aggravated these symptoms. I also think they need to be educated on the reasons why people go on contraceptives; I've often felt as though I've been put on one to stop pregnancy, when what I asked and what I was looking for was help with my endo*[metriosis]*/PCOS symptoms.* (22 year old regional resident)

*I have taken the Pill for four years and have always been overweight since first taking it. I was only told last week that people with a BMI over 35 shouldn't take the Pill because of an increased risk of blood clots. I have seen at least four different doctors in that time about filling my prescription for the Pill and none of them mentioned this. I am clearly overweight. Someone should have said something before now!* (22 year old regional resident)

### Women’s perspectives on the process of contraceptive discussions with doctors: what women experience

Women emphasised several aspects of the process of interacting with doctors as impacting on their ability and inclination to access contraception information via this source. In particular, some of the young women in this study reported that they found it difficult to speak to their doctor regarding contraception. Reasons for this varied between women and reflected both patient- and provider-related barriers. Some women described their youth as being an obstacle to comfortably having or actively seeking out a discussion about contraception with their doctor, while several reflected that personal discomfort restricted this discussion:

*I find it really difficult to talk to doctors as I am young and I feel that they don't think I take the information that they give me on board.* (23 year old urban resident)

*I feel embarrassed to talk to the doctor about the side-effects I am experiencing- no 23 year old should have no sex drive.* (23 year old urban resident)

*I find the Pill is quite unhelpful in the sense* [that] *when I take the brand I’m using, I find I’m very moody, stress more, and find it hard to get motivated! But I haven't spoken with any doctors about other contraception, one because I don’t have time and two,* [because] *I don’t feel too comfortable talking about other methods- but I will try!* (22 year old remote resident)

Furthermore, some women perceived their doctor to be unsupportive or even judgmental. Such perceptions appeared to act as barriers to women reporting satisfactory contraception consultations:

*I have found that when visiting the doctor regarding oral contraception, she was very abrupt and rude, provided hardly any information, and was reluctant to give me the prescription for the Pill. I have no medical issues, so there was no reason to say no. I find this to be quite rude and off-putting; many other people I know experience this as well. I am not sure if this is a cultural and age difference, but when going to the doctor on issues which are important, I expect that I am not treated in such a manner where I feel I am judged based on my desire to protect myself from pregnancy.* (22 year old urban resident)

*I find doctors can sometimes be shocked when I say I don’t want to use hormonal contraception. They are shocked and think that’s so irresponsible. But I’m 21- I’m a grown woman. To me, the side-effects of the Pill are so severe that I would personally rather take the chance of an unplanned pregnancy with my partner of two years than to continue to feel moody, depressed and anxious, with terrible skin and lactating breasts. There are so many judgments made by doctors about your choices, which are unacceptable.* (21 year old urban resident)

## Discussion

In this paper we examined the way that young women aged 18 to 23 years reported on interactions with doctors regarding contraception. Our analysis has suggested that several aspects of the patient-provider interaction are relevant for young Australian women accessing contraception, with emergent themes indicating that both the content and process of the contraception consultation are perceived by women as being important. These findings have a number of implications for clinical practice; in line with the patient-centred model of care, we present some recommendations below.

The desire to access comprehensive contraception-related information and a wide(r) range of contraceptive options through a doctor in order to be able to make an informed choice about the method most suitable for the individual was highlighted by the women’s narratives. This echoes themes emerging from a previous analysis of free-text comments from the Australian Longitudinal Study on Women’s Health (ALSWH), within which Australian women reported difficulties accessing information about contraception and barriers to method access [22]. Previous studies have demonstrated that a large proportion of women possess poor knowledge of contraception options [27, 28], in particular highly effective LARC methods [29], which acts as a barrier to their use- suggesting that doctors can play an active role in increasing (young) women’s awareness of contraceptive options. Indeed, in one recent study, participants articulated that knowledge about contraceptive options was seen by doctors as ‘assumed knowledge’ that women should possess [30], and a number of researchers have found that doctors do not comprehensively discuss contraceptive options with their patients [31, 32].

Acknowledging the time constraints faced by doctors in general practice settings [33], the utilisation of credible websites and/or decision-making tools for women may act as important adjuncts to the consultation proper. Such resources may enhance a woman’s understanding of evidence-based information to support her contraceptive choices, with the role of the doctor then being to determine her medical suitability for a particular method and to provide information about possible side-effects and their management within the consultation time. It also appears imperative to introduce more comprehensive education about contraception methods and options within health and physical education curricula in Australian schools, particularly if doctors are automatically assuming that women possess such knowledge [30].

Women in our study expressed a desire for consistent and accurate advice from their doctors, and reported frustrations upon encountering contradictory information, which led to uncertainty about their method options. Furthermore, women perceived their choice of method was restricted by their doctor, through the discussion of a limited number of options within the consultation time. Some women also expressed the belief that their young age was a factor biasing the method options discussed. This resonates with previous research, which found that providers hold biases with respect to the suitability of young and/or nulliparous women using certain contraceptive methods [31, 34, 35]. It is also recommended that doctors keep abreast of developments in contraceptive technology, access up-to-date guidelines with respect to the suitability for use and method contraindications for particular subsets of women, and refrain from making assumptions regarding the reasons that individual women are seeking contraception.

As described by some of our participants, contraceptive method choice may be influenced by non-contraceptive benefits; a finding comparable to data emerging from the Bettering the Evaluation and Care of Health (BEACH) program, which collected information about the clinical activities in general practice in Australia over an 18 year period [36]. These data suggest that over a quarter of contraceptive prescriptions for young women are made for non-contraceptive purposes, such as a treatment for acne or menstrual problems. In order for women to truly experience reproductive autonomy and control it is necessary that the contraceptive consultation be structured around a woman’s priorities, whether this be method effectiveness and/or other considerations [37]. Indeed, the contraceptive consultation is most effective when women’s concerns are understood by the healthcare provider [20].

A recent committee opinion by the American College of Obstetricians and Gynecologists recommends that doctors adopt a shared decision-making approach within the contraception consultation in order to optimise contraceptive choice [38]. That is, doctors should endeavour to take into account a woman’s unique circumstances and enquire as to the woman’s particular needs, preferences, expectations, and reproductive goals to ensure that advice is tailored appropriately [39]. Adopting such patient-centred, shared decision-making strategies could counteract the perception of some of our participants that their doctor was unsupportive or judgmental during the contraceptive consultation, which has previously been described by other studies conducted within both Australia [22] and overseas [21].

Women in our study emphasised the importance of discussing the possible side-effects of contraceptive methods with their doctor and voiced frustrations that they were not provided with (comprehensive) information about these. Discussing side-effects may be particularly relevant in women’s decision-making, with women consistently ranking side-effects among the three most important factors influencing their contraceptive choice [40]. Furthermore, the impact of side-effects on both physical and mental health has a considerable influence on a woman’s willingness to use or continue using certain methods [22, 41, 42]. Nevertheless, it has been demonstrated that effective pre-use discussion of side-effects can reduce discontinuation rates even if the woman experiences side-effects [43], and practitioners recognise the importance of providing more and better counselling in order to improve patient’s contraceptive use [44].

It is not merely the content but also the process of the contraception consultation that women perceived as important within our study. Some women reported difficulties discussing contraception-related topics with their doctor, attributing these difficulties to their young age, personal discomfort, and embarrassment with experiences of particular side-effects. Similar barriers have been reported in prior literature, with providers in one study reflecting that patients often exhibit discomfort talking about contraception [45]. Nonetheless, providers in that study also held the belief that patients should be the ones to initiate discussions about contraception. Given the discomfort reported by some women in our study, providers need to be aware that some women will find discussions about contraception uncomfortable and they may therefore need to employ a variety of communication strategies to support the woman in feeling comfortable to discuss sensitive contraception-related issues. Conducting an effective contraceptive consultation requires a combination of medical knowledge and communication skills, highlighting the need for doctors to receive good communication skills training and empower their patients to ask questions [39, 46].

In making these recommendations, we acknowledge the need to involve other health care providers, other than doctors, in educating, informing, and assisting women to make the best contraceptive choice for themselves. It is well within the scope of expertise of multidisciplinary healthcare providers to provide such information. Therefore, we propose a strategy to contraception provision that utilises other healthcare providers such as practice nurses, nurse practitioners, and pharmacists, so that the time-intensive nature of providing comprehensive contraception information can be shared. Another consideration may be to increase the capacity of practice nurses such that they are able to prescribe contraception as takes place in specialist sexual health services in the UK [47], for which there is current advocacy in Australia [48]. The scope is also expanding to include insertion of contraceptive implants and intrauterine devices (IUDs) [49]. Increasing access to contraceptive services in primary care, and professional education about contraception might also fill this gap in service provision.

There are very little data on the way in which doctors inform or advise women, and therefore affect decisions to start, continue, or cease a contraceptive method [12]. The gap between what (young) women want and what they experience when talking to their doctors about contraception will require further exploration in future studies.

### Study strengths, limitations, and suggestions for future research

The large number of responses that were available for analysis represents one of the strengths of this qualitative study and has enabled us to identify numerous aspects of the contraceptive consultation that are important for young women. Responses were provided by women responding to a general question about contraception in the context of a larger health survey, with women not specifically directed to consider the interactions that they had with their doctor nor their perception of contraceptive consultations; yet over 15% of women who answered the question felt that these were important enough to mention, giving weight to our data.

It must be acknowledged that the phrasing of the question- and indeed the focus of the survey in general- shaped the data collected, with participants possibly having been primed by the earlier questions to focus on particular aspects of their contraceptive experiences. As appears to be the nature of this kind of research, women who had problems with contraception were more likely to have wanted to write about it [25]; therefore, the overriding tone of the data presented here were reports of negative experiences. While it needs to be acknowledged that women were more likely to recall and recount negative experiences, it is nonetheless important to represent such occurrences and attempt to understand the reasons for them. It may be that using a direct approach to enquire about a woman’s interactions within contraceptive consultations would allow for a more balanced account to emerge, and clarification around points of interest (which was not possible in the current study) could be enhanced through utilising an interview format.

Free-text comments are increasingly recognised as a valid and rich source of data [50]. It is likely that the women wrote about their most salient and important experiences relating to contraception. Of course, this analysis was only able to capture such salient aspects of the contraceptive consultation from a woman’s perspective; in order to gain a comprehensive picture of what occurs in the contraceptive consultation, future research should also consider the contraceptive consultation from a practitioner’s viewpoint.

## Conclusions

This exploration has revealed the salient health communication issues for young Australian women when discussing contraception with doctors. Within the context of a contraception consultation, young women perceive both the content and process of the patient-provider interaction to be important. The data presented here provide a deeper understanding of how such consultations may be more constructively conducted within the framework of patient-centred care, with the recommendation for doctors to adopt shared decision-making strategies within practice. It is important for all women to have comprehensive contraceptive options presented to them and be supported in making choices about contraception that suit their individual circumstances.

## Abbreviations

ALSWH: Australian Longitudinal Study on Women’s Health
BEACH: Bettering the Evaluation and Care of Health program
BMI: Body mass index
CUPID: Contraceptive Use, Pregnancy Intentions and Decisions survey
IUD: Intrauterine device
IUS: Intrauterine system
LARC: Long-acting reversible contraception
LGN-EC: Levonorgestrel emergency contraceptive pill
OCP: Oral contraceptive pill
PCOS: Polycystic ovarian syndrome

## Footnotes

1 Geographical location is defined here in accordance to the Australian Statistical Geography Standard [51].

2 A contraceptive implant is a rod-shaped etonogestrel-releasing implant that is inserted subdermally in the upper inner arm to prevent pregnancy for up to three years. Tradenames: Implanon/Implanon NXT.

3 A hormonal intrauterine device (IUD), also known as an intrauterine system (IUS), is a T-shaped levonorgestrel-releasing device placed in the uterus to prevent pregnancy for up to five years. Tradename: Mirena.

4 A vaginal ring is an etonogestrel/ethinyl estradiol-releasing ring that is placed in the vagina for three weeks then replaced with a new one a week later. Tradename: NuvaRing.
